# Chronic post-traumatic intramedullary lesions in dogs, a translational model

**DOI:** 10.1371/journal.pone.0187746

**Published:** 2017-11-22

**Authors:** Neringa Alisauskaite, Ingo Spitzbarth, Wolfgang Baumgärtner, Peter Dziallas, Sabine Kramer, Ricarda Dening, Veronika Maria Stein, Andrea Tipold

**Affiliations:** 1 Department of Small Animal Medicine and Surgery, University of Veterinary Medicine Hannover, Hannover, Germany; 2 Department of Pathology, University of Veterinary Medicine Hannover, Hannover, Germany; 3 Centre for Systems Neuroscience, University of Veterinary Medicine Hannover, Hannover, Germany; Faculty of Animal Sciences and Food Engineering, University of São Paulo, BRAZIL

## Abstract

**Objectives:**

Post-traumatic intramedullary myelopathies and cavitations are well described lesions following spinal cord injury (SCI) in humans and have been described in histopathological evaluations in dogs. Human intramedullary myelopathies/cavitations are associated with severe initial SCI and deterioration of clinical signs. Canine intervertebral disc extrusions share similarities with SCI in humans. In this descriptive study, magnetic resonance imaging (MRI) findings in spinal cords of dogs suffering from chronic post-traumatic myelopathies, including cavitations, are elucidated. An additional aim of the study was to compare diagnostic imaging and histopathological findings and identify similarities between human and canine chronic post-traumatic spinal cord lesions.

**Methods:**

Thirty-seven dogs with thoracolumbar SCI and one or more 3Tesla MRI investigations more than 3 weeks after SCI were included. Extent of intramedullary lesions and particularly cavitations were evaluated and measured in sagittal and transverse MRI planes. These data were compared with clinical data.

**Results:**

A total of 91.9% of study patients developed chronic intramedullary lesions, and 86.5% developed intramedullary cavitations. Paraplegia without deep pain perception at initial examination was significantly associated with longer chronic myelopathies/cavitations (P = 0.002/P = 0.008), and with larger maximal cross-sectional area (mCSA) of the lesions (P = 0.041/0.005). In addition, a non-ambulatory status after decompressive surgery was also associated with the development of longer intramedullary lesions/cavitations (P<0.001) and larger lesion mCSA (P<0.001/P = 0.012). All dogs with negative outcome developed myelopathies/cavitations. In the group of 21 dogs with positive outcome, 3 did not develop any myelopathies, and 5 did not develop cavitations.

**Conclusions:**

Development of chronic intramedullary lesions/cavitations are common findings in canine SCI. Extensive chronic intramedullary lesions/cavitations reflect a severe initial SCI and negative clinical outcome. This supports the hypothesis that chronic spinal cord changes following SCI in humans share similarities with canine chronic spinal cord changes after spontaneous intervertebral disc extrusion.

## Introduction

Human and canine intervertebral disc herniation (IVDH) and the consequent spinal cord pathology differ extensively. Disc protrusions are common in humans [1]. In the thoracic area, symptomatic IVDHs are rare [2–4]. In the lumbar area, they commonly occur at the level of L4-L5 and L5-S1, where peripheral nerves are affected, causing milder clinical symptoms [2–4]. In contrast, IVD extrusions are common in dogs, causing contusive and compressive lesions of the spinal cord resembling spinal cord injuries (SCI) after external trauma in humans, making the dog a suitable animal model for SCI [5–7].

The most commonly seen chronic intramedullary pathologies in histopathological studies of post-traumatic SCI include spinal cord degeneration, atrophy, demyelination, gliosis, malacia, necrosis and subsequent cavitations [5, 8–12].

Non-cavitational myelopathies in human studies are recognised as hyperintense ill-defined intramedullary areas with a less intense signal in comparison to cerebrospinal fluid (CSF) signal in T2 weighted magnetic resonance images (T2W MRI) [13–17]. These areas are described as hypointense or isointense areas in T1 weighted images (T1WI) [13–16]. In canine patients with chronic spinal cord changes, MRI features of non-cavitational intramedullary lesions have been poorly described. Few studies have observed intramedullary hyperintense signals in T2WI MR images of dogs with chronic paraplegia after acute IVDH [18, 19].

In addition to the described myelopathies, cavitation and/or syringomyelia might be observed. In human MRI studies, syringomyelia is defined as a tubular CSF signal intensity [14, 17]. The distinction between post-traumatic cavitations and syringomyelia is cumbersome, but most authors agree that cavitations are more restricted to the SCI epicentre lesions, while syringomyelia extends beyond the limits of SCI [14, 16, 17]. Intramedullary cavitations and/or syringomyelia in MRI of dogs as well as in humans are seen as hyperintense intraparenchymal signals with well-defined margins (isointense or slightly hypointense to CSF signal) in T2WI and hypointense in T1WI [20, 21].

The incidence of post-traumatic chronic intramedullary changes and intramedullary cavitations/syringomyelia in human patients is still uncertain, but is reported to reach up to 80% and 51%, respectively [14, 22, 23]. Unsuccessful outcomes after acute IVDH have been observed to be associated with severe chronic histopathological changes in the spinal cord parenchyma of canine patients [5, 8, 9, 11]. However, dogs that underwent control MRI examinations after successful IVDH treatment had no observable changes in MR images of their spinal cords [24]. Hyperintensity in T2WI in the initial MRI of dogs suffering from acute intervertebral disc herniations is well described and is associated with a worse prognosis for the patient [25, 26]. However, the features of chronic post-traumatic intramedullary changes, including post-traumatic syringomyelia in MRI have not yet been described in dogs.

It has been observed in human patients that myelopathies and cavitations/syringomyelia are associated with severity of initial clinical signs [14–16, 27], post-traumatic recovery [14, 16] and spinal canal compromise [27].

The aim of the study was to describe chronic intramedullary lesions detectable in MR images following SCI in dogs and to compare the features of the intramedullary lesions with the clinical data. The hypotheses were that extensive myelopathies/cavitations are associated with severe initial SCI, negative outcome and higher number of manipulations on the spinal cord. Therefore, the extent of chronic intramedullary lesions/cavitations was compared with the initial neurological status and the presence of intramedullary hyperintensity in initial MR images. In addition, the extent of myelopathies/cavitations was compared to the ambulation status of the patients following treatment and the amount of decompressive surgeries performed. Spinal cord atrophy was hypothesised to be related to negative outcome of the patients. Therefore, the spinal cord diameter reduction in the lesion epicentre was compared to the outcome.

## Materials and methods

### Clinical settings

The database of the Small Animal Clinic of the University of Veterinary Medicine Hannover from 2010 to 2014 was reviewed to identify canine patients with chronic SCI. The following inclusion criteria were used to select the patients: body weight less than 20 kg, history of thoracolumbar spinal cord injury (mainly IVDH), which has been confirmed with MRI, paraplegic state at presentation in the veterinary hospital with present or absent deep pain perception (grade IV or V [28]), 3Tesla MRI investigations performed 3 weeks or later following spinal cord trauma. All patients with further lesions in their spinal cords, with the exception of mild intervertebral disc protrusions, were excluded from the study.

Part of the recruited animals were involved in another study (the permission was obtained from the Lower Saxony State Office for Consumer Protection and Food Safety, File Number 33.9-42502-04-11/0661), and other animals were retrospectively searched in the database of the Small Animal Medicine and Surgery Department of the University of Veterinary Medicine in Hannover. Dogs were anaesthetised and/or euthanised according to ethical principles using different medications, including acepromazine, levomethadone, propofol, isoflurane and pentobarbital. All efforts were made to minimise suffering. Approval was obtained from all dog owners to perform the study. An additional permission from the State Office for Consumer Protection and Food Safety was not applicable for the project, because of the retrospective origin of the study.

Based on the severity of neurological dysfunction, neurological examination results were summarised as Grades I, II, III, IV or V [28]:

Grade I spinal hyperesthaesia only
Grade II ambulatory paraparesis
Grade III non-ambulatory paraparesis
Grade IV paraplegia with present deep pain perception
Grade V paraplegia with absent deep pain perception

Information about neurological grade immediately after SCI was not available for 10 patients because they were presented to veterinarians who referred the cases only in the subacute or chronic stage of the disease (Tables 1 and 2). All 10 of these patients were non-ambulatory at the presentation to the referring veterinarians (Grade III-V).

**Table 1:** Signalment, clinical and initial imaging data of patients with negative outcome.

Patient ID	Age (years)	Breed	Sex	Weight (kg)	Neurological grade immediately after SCI	Initial spinal cord injury location (vertebral level)	Hyperintensity in initial MRI	Number of decompressive surgeries	Time interval between SCI and adequate decompression (days)
1	5	Dachshund	M	9.1	n.a.	T11-T12	n.a.	2	90
2	3	Dachshund	F	6.7	5	T11-T12; T12-T13	Yes	2	2
3	3	Mix	M	7.2	n.a.	T12-T13; T13-L1; L1-L2; L2-L3	n.a.	1	1
4^1^	2	Mix	M	5.8	n.a.	L3-L4	n.a.	1	60
5	6	Dachshund	F	8.2	n.a.	T12-T13	n.a.	1	1
6	4	French bulldog	M	16	n.a.	T12-T13	n.a.	2	2
7^2^	2	French bulldog	cF	12.8	n.a.	L2-L3	n.a.	1	2
8	8	Dachshund	F	8.8	5	T11-T12	Yes	1	1
9	5	Dachshund	F	10.5	5	T12-T13	Yes	2	1
10	3	Mix	M	2.5	n.a.	T13-L1; L1-L2	n.a.	1	1
11^2^	4	Dachshund	M	3.5	n.a.	T13-L1	n.a.	1	1
12	4	Dachshund	cM	5.2	4	L2-L3	n.a.	1	200
13	4	Mix	cF	5.2	5	T13-L1	n.a.	1	1
14	11	Dachshund	cM	7.5	4	T13-L1; T12-T13; L1-L2	Yes	2	2
15	4	Dachshund	cF	11.5	5	T12-T13; T13-L1; L1-L2	Yes	2	1
16^2^	5	Jack Russel Terrier	M	10.1	5	L2-L3	Yes	1	1

^1^ Patient had L3-L4 vertebral fracture.

^2^ Patients died or were euthanised within 11 months following SCI.

n.a.–data not available.

M–male, cM–castrated male, F–female, cF–castrated female.

**Table 2:** Signalment, clinical and initial imaging data of patients with positive outcome.

Patient ID	Age (years)	Breed	Sex	Weight (kg)	Neurological grade immediately after SCI	Initial spinal cord injury location (vertebral level)	Hyperintensity in initial MRI	Number of decompressive surgeries	Time interval between SCI and adequate decompression (days)
**17**	7	Mix	cM	14	4	T13-L1	yes	1	1
**18**	6	Chihuahua	F	4.5	4	T12-T13; T13-L1	yes	1	1
**19**	7	Dachshund	cM	12.2	4	T13-L1	yes	1	1
**20**	2	Shi Tzu	F	5.6	5	T12-T13; T13-L1	yes	1	1
**21**	6	Dachshund	cF	7.6	n.a.	T11-T12	n.a.	2	1
**22**	2	French bulldog	F	11.5	4	T12-T13; T13-L1	yes	1	1
**23**	9	Dachshund	M	14.8	4	L1-L2	yes	2	1
**24**	5	Dachshund	M	8.9	5	T13-L1	no	2	2
**25**	6	Dachshund	F	8.6	5	T12-T13	no	2	1
**26**	5	Dachshund	cF	5.8	4	T12-T13; L1-L2	no	1	1
**27**	4	Jack Russel Terrier	cM	8	4	T12-T13	yes	1	1
**28**	3	French bulldog	M	13.4	4	L1-L2; L2-L3; T12-T13	yes	1	1
**29**	7	Bolonka Zwetnaya	M	8.7	4	T13-L1; L2-L3	no	1	1
**30**	10	Dachshund	F	6.5	4	T11-T12	no	1	1
**31**	10	Dachshund	cF	8.5	n.a.	T13-L1	n.a.	1	21
**32**	3	Mix	cM	6.4	5	L1-L2	yes	2	1
**33**	5	Lhasa Apso	cF	11	4	L2-L3	no	1	1
**34**	2	Small Munsterlander	cM	13.5	4	T12-T13	no	1	1
**35**	4	Jack Russel Terrier	cM	8.3	4	L1-L2	no	1	1
**36**	6	Dachshund	cM	12.7	4	T10-T11	yes	2	1
**37**	10	Mix	cF	19.6	4	T13-L1	yes	1	1

n.a.–data not available.

M–male, cM–castrated male, F–female, cF–castrated female.

All clinical data were reviewed, including age, breed, sex, body weight, clinical history, information about applied treatment and outcome of the patients. Follow-up was available for at least 7 months after SCI, except in the patients that died or were euthanised upon request of the owners (Table 1).

The time interval between onset of paraplegia and adequate decompression of the spinal cord was noted (Tables 1 and 2). Decompression was considered adequate when MRI observers subjectively agreed that the spinal cord was not compromised.

The number of decompressive surgeries performed on study patients, including the ones performed by other veterinary facilities, was recorded. The outcome was grouped into two categories:

- Negative—animals did not regain ambulation within 7 months after presentation in the hospital or were euthanised/died spontaneously without showing signs of neurological improvement;
- Positive–animals returned to ambulatory state within 7 months following SCI (≥grade II).

MRI examinations were performed using a 3.0T Philips Achieva MRI scanner (Phillips Medical Systems, Eindhoven, Netherlands) in all patients, and a 15-channel SENSE (sensitivity encoding) spine coil was applied. Spin echo T1W transverse (TR = 491.6, TE = 8, slice thickness = 2 mm), T2W transverse (TR = 8418.8, TE = 120, slice thickness = 2 mm), T2W FLAIR transverse (TR = 10000, TE = 140, slice thickness = 3.5 mm) and T2W sagittal (TR = 3100, TE = 120, slice thickness = 1.8 mm) images were acquired.

Decompressive surgeries were performed in all dogs, in which spinal cord compression was confirmed in diagnostic imaging. To decompress the spinal cord, a standard hemilaminectomy was performed using the paramedian approach to the facet joints and accessory processes of the vertebra [28].

### Imaging data analysis

MRI images were analysed, and measurements were made in sagittal and transverse T2W, T1W and FLAIR MRI views using measurement and marking tools installed in the EasyImage image processing software (EasyImage®, Hannover, Germany). MR images were reviewed by two board certified neurologists (A.T. and V.S.).

All 37 patients had T2W images available in all MR investigations. Other available sequences are listed in Table 3. The initial SCI location (epicentre) in MRI was defined as the location of IVDH and spread disc material compressing the spinal cord. In 26 dogs, acute intramedullary changes in the spinal cord could be retrospectively evaluated on initial MRI examinations performed immediately (≤48 hours) after SCI.

**Table 3:** Time points of MRI investigations following SCI.

Patient ID	Time point of presentation at the clinic in regard to SCI	MRI sequences available^1^	MRI time points following SCI							
			3 weeks– 2 months	2–5 months	5–8 months	8–11 months	11–14 months	14–17 months	17–20 months	>20 months
1	>48 hours	T2, T1, FLAIR		xx	x	X				
2	>48 hours	T2, T1, FLAIR		x	x	X				
3	>48 hours ^2^	T2, T1, FLAIR				x	xx	X		
4	>48 hours	T2, T1		xx	x	X				
5	>48 hours^2^	T2, T1, FLAIR			xx	x	X			
6	>48 hours^2^	T2, T1, FLAIR					xx	X	X	
7	>48 hours^2^	T2, T1				x X				
8	≤48 hours	T2, T1, FLAIR	x	x	x X					
9	≤48 hours	T2, T1, FLAIR	x	x	X					
10	>48 hours^2^	T2, T1, FLAIR					xx	X	X	
11	>48 hours^2^	T2, T1, FLAIR		x	x	x	X			
12	>48 hours	T2, T1, FLAIR			x	x	x	X		
13	>48 hours^2^	T2, T1, FLAIR	x	xx		X				
14	≤48 hours	T2, T1, FLAIR	x	x	X					
15	≤48 hours	T2, T1, FLAIR	x	x	X					
16	≤48 hours	T2, T1, FLAIR	x	X						
17	≤48 hours	T2, T1, FLAIR		X						
18	≤48 hours	T2, T1, FLAIR	x	x		X				
19	≤48 hours	T2, T1, FLAIR		x	X					
20	≤48 hours	T2, T1, FLAIR		X						
21	>48 hours^2^	T2, T1, FLAIR	x		X					
22	≤48 hours	T2, T1, FLAIR		X						
23	≤48 hours	T2, T1, FLAIR		X						
24	≤48 hours	T2, T1, FLAIR	x	X						
25	≤48 hours	T2, T1		X						
26	≤48 hours	T2, T1		X						
27	≤48 hours	T2, T1		X						
28	≤48 hours	T2, T1, FLAIR	x	x	X					
29	≤48 hours	T2, T1		X						
30	≤48 hours	T2								X
31	>48 hours	T2, T1, FLAIR		X						
32	≤48 hours	T2, T1, FLAIR		X						
33	≤48 hours	T2, T1, FLAIR		X						
34	≤48 hours	T2, T1, FLAIR		X						
35	≤48 hours	T2, T1, FLAIR		X						
36	≤48 hours	T2, T1, FLAIR		X						
37	≤48 hours	T2, T1		X						

T2—T2 weighted MR images, T1—T2 weighted MR images, FLAIR–Fluid-attenuated inversion recovery MR images.

x—MRI examination performed once in the time interval, xx—MRI examination performed twice in the time interval, X–time point of the last MRI examination available, which was used for comparison with clinical data.

^1^Mentioned sequences were available in at least 1 MRI examination.

^2^Dogs had decompressive surgery in other veterinary clinics or hospitals before presentation in the Small Animal Clinic of the University of Veterinary Medicine in Hannover.

Chronic intramedullary changes were assessed in each study patient using MRI investigations performed 3 weeks or later following SCI. The time of MRI investigations in regard to acute SCI was recorded. Time points of follow-up MRI examinations of chronic lesions varied widely among the patients and were divided into 9 groups for simplification (Table 3).

**Fig 1:**
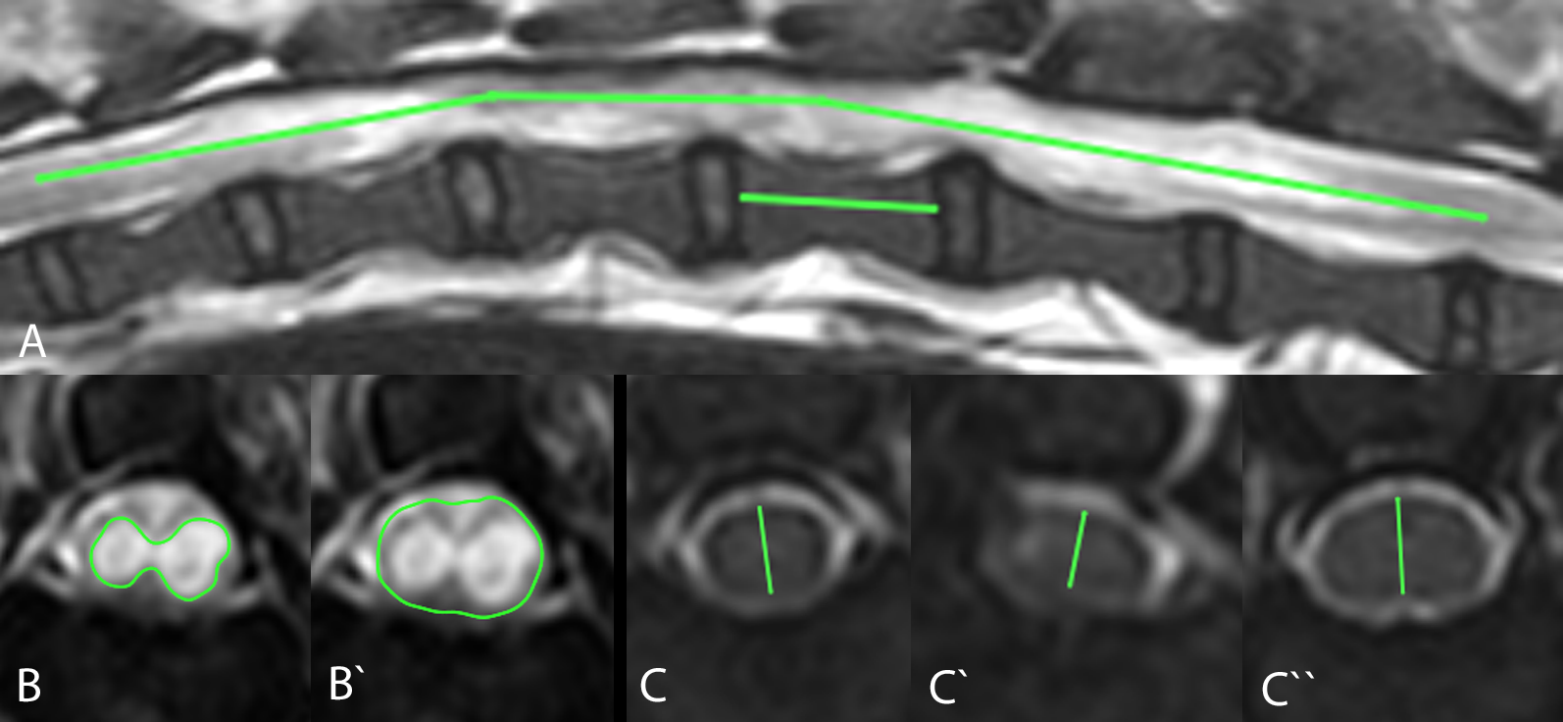
Transverse T2W and T1W MR images of intramedullary lesions/cavitations at the level of the SCI epicentre in the 3 dogs that were subsequently submitted for post-mortem histopathological examination. Note the intramedullary hyperintense signal (arrow) in T2W (A, B) and hypointense signal in T1W MR images (A’, B’) in comparison to normal spinal cord parenchyma in the spinal cords of the French bulldog (A, A': patient ID 7) and dachshund (B, B': patient ID 11) 3 months following SCI. These signal changes are compatible with fluid-filled intramedullary cavitations, as they are isointense to the CSF signal. More subtle changes were observed in a Jack Russel terrier (C, C': patient ID 16) spinal cord 6 months following SCI (C). In some areas, the intramedullary signal is hyperintense in comparison to the normal spinal cord signal in T2W MR images, but it is hypointense in comparison to the CSF signal (arrowhead). Corresponding areas look isointense to spinal cord in T1W MR images (C’).

Intramedullary cavitations were defined as a well demarcated hyperintense (equal to CSF or slightly lower) signal in T2WI and hypointense signal in T1WI and/or FLAIR images in spinal cord parenchyma. Post-traumatic syringomyelia was considered a cystic intramedullary lesion extending beyond the margins of the SCI epicentre [14, 16, 17]. Intramedullary lesions were defined as all abnormal intraparenchymal spinal cord signal intensities in MRI. Such areas included non-cavitational myelopathies (ill-demarcated lesions, hyperintense in T2WI in comparison to surrounding spinal cord parenchyma signal, but less signal intensity than surrounding CSF) and well demarcated cavitations as described above (Fig 1).

In all MRI examinations 3 weeks post-SCI or later, the following features were investigated: SCI location (epicentre), presence of intramedullary lesions, presence of intramedullary cavitations, location of intramedullary lesions in regard to SCI epicentre (cranially, caudally, both directions or restricted to SCI epicentre), length of intramedullary lesions and cavitations in sagittal MRI views, maximal cross-sectional area (mCSA) of spinal cord lesion in transverse views and degree of spinal cord diameter reduction.

**Fig 2:**
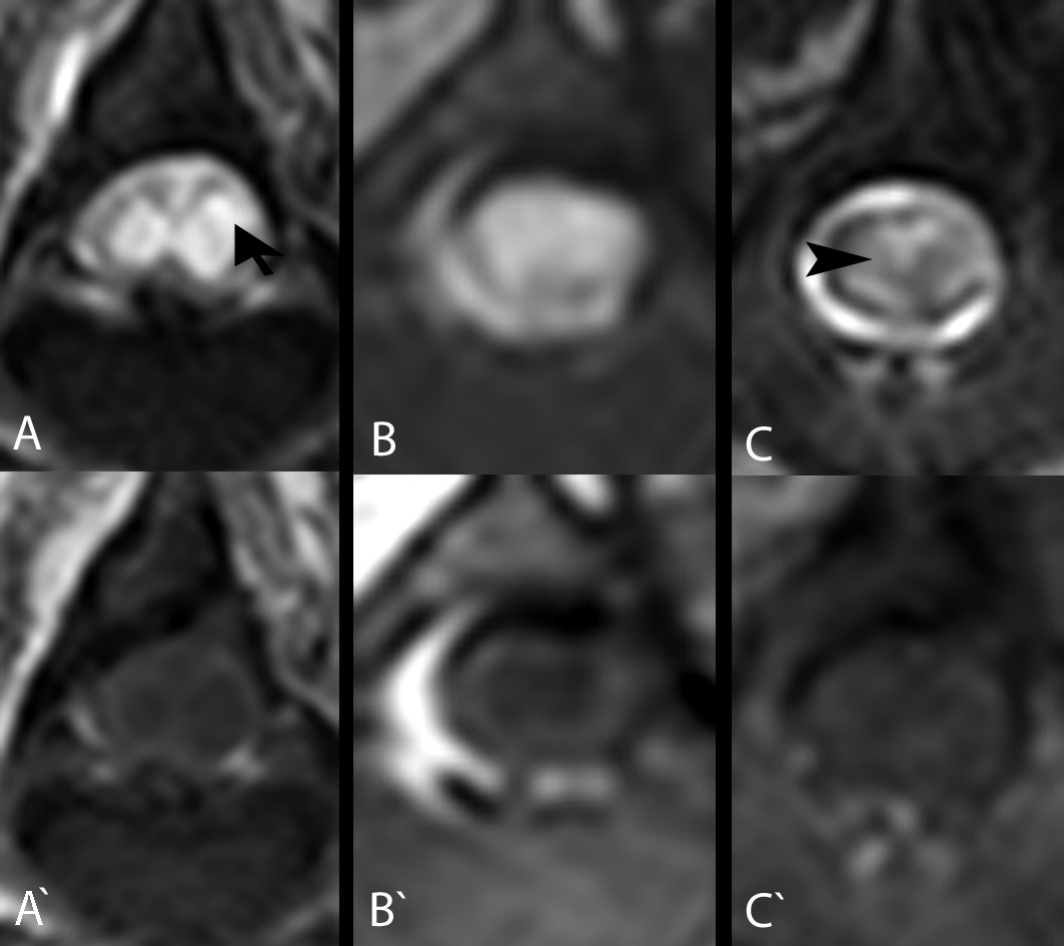
Measurement of the length, maximal cross-sectional area (mCSA) of intramedullary lesions/cavitations and spinal cord diameter at the lesion epicentre. The length of intramedullary lesions/cavitations was estimated by measuring the length of the intramedullary lesions/cavitations in T2W MR images and calculating the ratio to the length of the L2 vertebra (A). Maximal CSA of spinal cord lesions/cavitations was measured by measuring the area of the spinal cord lesion/cavitation in the T2W MR images (B) and dividing it by the area of the spinal cord at the same level (B‘), which led to a percentage of the damaged spinal cord. Spinal cord diameter was measured in T2W MRI at the lesion epicentre (C ‘) and at the level of the normal spinal cord cranially (C) and caudally (C ‘‘). The difference of diameters led to the estimated reduction of spinal cord diameter.

To measure the extent of intramedullary lesions, we used the methods described previously [29]. The length of intramedullary lesions and intramedullary cavitations were compared to the length of second lumbar (L2) vertebral body length [30]. The L2 vertebral body was measured in sagittal T2WI from cranial to caudal end plates [26]. The extent of intramedullary lesion/cavitation was divided by the length of the L2 vertebral body and expressed as a number (Fig 2):

Lesionlength(n)=Lengthofthelesion(mm)/L2vertebralbodylength(mm)

The mCSA of spinal cord lesions in transverse MRI views was calculated by dividing the area of affected spinal cord parenchyma by the area of the whole spinal cord at the same level and multiplying by 100 (expressed in percentage) (Fig 1). For simplification, the mCSA was categorised as no lesion, mild (<35%), average (35–75%) and severe (75–100%) lesion.

$$\mathrm{mCSA}\;\left(\%\right)=\mathrm{Lesion}\;\mathrm{area}\;(mm^{2})\;/\;\mathrm{Spinal}\;\mathrm{cord}\;\mathrm{area}\;(mm^{2})\;\times \;100$$

The diameter of the spinal cord in the SCI epicentre was measured in transverse MRI views in all dogs with chronic SCI and compared with the diameters of apparently normal spinal cord diameter cranially and caudally of the lesion (1 spinal cord segment cranially and caudally of the lesion epicentre in most cases) and expressed by number as normal (1–0.95), mildly (0.75–0.95), moderately (0.35–0.75) or severely (0–0.35) reduced (Fig 1) [26]. If the diameter of spinal cord fell into different categories in comparison with apparently normal spinal cord in the segment cranially and caudally, the higher category was chosen for evaluation of results. The following formula was used to express the degree of spinal cord diameter reduction at the SCI epicentre:

Spinalcorddiameterreductiondegree(n)=DiameterinSCIepicentre(mm)/Diametercranially(caudally)toSCIepicentre(mm)

Two study patients died spontaneously at home, and 1 dog had to be euthanised on request of the owners. With the owners’ consent these dogs underwent routine necropsy at the Department of Pathology of the University of Veterinary Medicine Hannover. During necropsy, tissue samples of multiple organs, including the spinal cord, were collected and fixed in 10% non-buffered formalin, followed by embedding in paraffin wax. Three micrometre-thick serial sections of the spinal cord at the site of the epicentre were stained with haematoxylin and eosin and underwent histopathological examination. Pictures of the sections were taken with a Keyence Microscope (BZ-9000, Keyence Deutschland GmbH, Neu-Isenburg, Germany) and adjusted for contrast and brightness, if necessary. Histopathological changes were compared to MRI results.

### Statistical analysis

Data obtained in the MRI investigations were compared to clinical data utilising different statistical tests (Student's t-test, chi-squared test and McNemar's tests) using the IBM® SPSS® Statistics software Version 23 (IBM Corp., USA). If multiple MRIs were performed on a patient at different time points, the latest one was used for the comparison with the clinical data. To test for associations between the extension of the lesions in sagittal views and qualitative clinical data, including hyperintense intramedullary signal in T2WI in initial MRI, number of decompressive surgeries, outcome and neurological grade before surgery, the Student's t-test was used. To determine the relationship between clinical data and the mCSA of lesions observed in transverse MRI views and degree of reduction of spinal cord diameter, the chi-squared test was used. On paired nominal data, such as positive/negative outcomes, and intramedullary lesions restricted to the SCI epicentre/extended lesions, McNemar's test was performed.

## Results

Thirty-seven dogs fulfilled the inclusion criteria, 36 of which suffered from IVDH and 1 from a L3-L4 vertebral fracture. Twelve of f the 37 patients (32.4%) were diagnosed and treated in other veterinary facilities and were presented at the Small Animal Clinic of the University of Veterinary Medicine Hannover more than 48 hours after onset of paraplegia. Twenty-five of the 37 dogs (67.6%) were presented in the hospital within 48 hours following SCI.

Information about neurological grade at presentation, age, gender and breed distribution among study dogs, and number of decompressive surgeries performed on the patients are presented in Tables 1 and 2. Twenty-five of the 37 patients (67.6%) had 1 decompressive surgery, and 12 of the 37 (32.4%)–had 2 decompressive surgeries.

Outcomes were negative in 16 of the 37 (43.2%) study patients and positive in 21 of 37 (56.8%, Table 4). Three dogs, which were categorised as negative outcome patients, died within 11 months after SCI. One of them was euthanised 3 months after SCI on request of the owner, because of lack of improvement, and 2 died due to reasons not related to SCI 11 and 10 months post-SCI. The time interval between acute onset of paraplegia and adequate decompression ranged from 1 to 200 days (Tables 1 and 2). Hemilaminectomies were performed on 28 of the 37 (75.7%) patients within 24 hours after SCI, on 5 of 37 (13.5%) between 24 hours and 48 hours following SCI and on 4 of 37 (10.8%) later than 48 hours after SCI (Table 3).

**Table 4:** Chronic intramedullary lesion, cavitation, mCSA and SCA distribution among patients with positive and negative outcome and initial neurological grades IV and V.

	Chronic IL	Chronic IC	Chronic IL extending beyond SCI epicentre	Post-traumatic syringomyelia	Length of IL (median, range)	Length of IC (median, range)	Mild mCSA		Moderate mCSA		Severe mCSA		SCA
							IL	IC	IL	IC	IL	IC	
**Positive outcome**	18/21	16/21	9/21	6/21	1.66/4.66	0.36/2	13/21	12/21	4/21	3/21	1/21	1/21	16/21
**Negative outcome**	16/16	16/16	13/16	12/16	5.32/10.35	4.22/9.39	2/16	6/16	4/16	8/16	10/16	2/16	16/16
**Grade IV**	13/16	11/16	5/16	4/16	1.60/4.92	0.29/4.38	11/16	10/16	1/16	0/16	1/16	1/16	11/16
**Grade V**	10/10	10/10	8/10	6/10	4.01/8.09	1.70/3.77	4/10	5/10	3/10	4/10	3/10	1/10	10/17

IL–intramedullary lesions, IC–intramedullary cavitations, mCSA–maximal cross-sectional area, SCA–spinal cord atrophy.

Imaging data immediately after SCI (≤48 hours) was available in 25 patients, and an intramedullary hyperintensive signal in T2WI in the initial MR examinations was detected in 18 of 25 (72%) dogs (Tables 1 and 2).

Thirty-four of 37 (91.9%) study patients developed chronic intramedullary lesions, and 32 of 37 (86.5%) developed intramedullary cavitations. Median values of length of intramedullary lesions and cavitations were 3.17 and 1.24, respectively.

Lesion distribution in regard to lesion epicentre is illustrated in Tables 5 and 6.

**Table 5:** Initial SCI location, length and location of chronic intramedullary lesions/cavitations, mCSA of intramedullary lesions/cavitations and severity of spinal cord atrophy in patients with negative outcome.

Patient ID	Initial SCI location	Length of intramedullary lesions (ratio with L2 vertebral length)	Length of intramedullary cavitations (ratio with L2 vertebral length)	Length of intramedullary lesions (vertebral level)	Length of intramedullary cavitations (vertebral level)	Maximal CSA of intramedullary lesions	Maximal CSA of intramedullary cavitations	SCA
**1**	T11-T12	5.04	4.6	T11-L2	T11-L2	severe	mild	mild
**2**	T11-T12; T12-T13	4.9	3.4	T11-L3	T11-L1	moderate	moderate	moderate
**3**	T12-T13; T13-L1; L1-L2; L2-L3	7.23	6.96	T11-L4	T11-L4	severe	severe	moderate
**4**^**1**^	L3-L4	8.52	7.33	T10-L4	T10-L4	moderate	mild	-
**5**	T12-T13	5.6	5.6	T11-L2	T11-L2	severe	mild	mild
**6**	T12-T13	10.72	9.76	T11-L3, T5-T10, T3-T4	T12-L2, T5-T10, T3-T4	severe	moderate	mild
**7**^**2**^	L2-L3	7.6	6.02	T12-L4	T13-L4	severe	moderate	-
**8**	T11-T12	3.31	1.39	T10-T13	T11-T12	moderate	moderate	mild
**9**	T12-T13	4.98	3.61	T11-L2	T11-L2	mild	mild	mild
**10**	T13-L1; L1-L2	4.65	3.74	T13-L3	T13-L3	severe	moderate	moderate
**11**^**2**^	T13-L1	10.78	8.57	T9-L4	T10-L3	severe	moderate	mild
**12**	L2-L3	2.02	1.64	L2-L3	L2-L3	mild	mild	moderate
**13**	T13-L1	8.62	4.05	T10-L4	T12-L1; L3	severe	moderate	mild
**14**	T13-L1; T12-T13; L1-L2	4.92	4.38	L1-L4	L1-L4	severe	severe	moderate
**15**	T12-T13; T13-L1; L1-L2	5.7	3.78	T12-L3	T12-L2	severe	moderate	moderate
**16**^**2**^	L2-L3	0.53	0.37	L3	L3	moderate	mild	moderate

SCI–spinal cord injury, maximal CSA (mCSA)–maximal cross-sectional area, SCA–spinal cord atrophy. Mild mCSA of lesions—<35%, average—35–75% and severe– 75–100% of spinal cord diameter. Mild SCA—0.75–0.95, moderate—0.35–0.75 and severe–<0.35 in regard to unaffected spinal cord diameter cranially/caudally from lesion epicentre.

**Table 6:** Initial SCI location, length and location of chronic intramedullary lesions/cavitation, mCSA of intramedullary lesions/cavitations and severity of spinal cord atrophy in patients with positive outcome.

Patient ID	Initial SCI location	Length of intramedullary lesions (ratio with L2 vertebral length)	Length of intramedullary cavitations (ratio with L2 vertebral length)	Length of intramedullary lesions (vertebral level)	Length of intramedullary cavitations (vertebral level)	Maximal CSA of intramedullary lesions	Maximal CSA of intramedullary cavitations	SCA
**17**	T13-L1	0.83	0.83	T13-L1	T13-L1	moderate	moderate	mild
**18**	T12-T13; T13-L1	2.09	0.41	T12-L1	T13	mild	mild	mild
**19**	T13-L1	2.08	0	T13-L1	-	mild	-	mild
**20**	T12-T13; T13-L1	3.35	0.28	T12-L1; L2	T12-L1; L2	mild	mild	mild
**21**	T11-T12	2.86	0.68	T9-T12	T11-T12	moderate	moderate	severe
**22**	T12-T13; T13-L1	1.05	1.05	T13; L2	T13; L2	mild	mild	-
**23**	L1-L2	0.06	0.06	L1	L1	mild	mild	mild
**24**	T13-L1	3.17	2	T13-L2	L1-L2	mild	mild	moderate
**25**	T12-T13	4.66	1.26	T11-L2	T12; L2	mild	mild	mild
**26**	T12-T13; L1-L2	0	0	-	-	-	-	-
**27**	T12-T13	0.21	0.21	T12	T12	mild	mild	-
**28**	L1-L2; L2-L3; T12-T13	3.3	1.68	L1-L3	L1-L2	mild	mild	-
**29**	T13-L1; L2-L3	0	0	-	-	-	-	mild
**30**	T11-T12	0	0	-	-	-	-	-
**31**	T13-L1	0.76	0.38	T13-L1	T13-L1	moderate	moderate	mild
**32**	L1-L2	1.85	1.24	T13-L2	L1-L2	severe	severe	mild
**33**	L2-L3	1.53	0.33	L2; L3-L4	L2; L4	mild	mild	mild
**34**	T12-T13	2.04	0.39	T12-T13	T12	mild	mild	mild
**35**	L1-L2	1.66	0.25	L1-L2	L2	mild	mild	mild
**36**	T10-T11	1.51	0.46	T11-T12	T11; T12	moderate	mild	mild
**37**	T13-L1	4	0	T13-L3	-	mild	-	mild

SCI–spinal cord injury, maximal CSA (mCSA)–maximal cross-sectional area, SCA–spinal cord atrophy. Mild mCSA of lesions—<35%, average—35–75% and severe– 75–100% of spinal cord diameter. Mild SCA—0.75–0.95, moderate—0.35–0.75 and severe–<0.35 in regard to unaffected spinal cord diameter cranially/caudally from lesion epicentre.

Maximal CSA of intramedullary lesions/cavitations was measured in transverse MR images. Intramedullary lesions were mild (<35%) in 15 of 37 (40.5%) patients, average (35–75%) in 8 of 37 (21.6%) and severe (75–100%) in 11 of 37 (29.7%). Intramedullary cavitations were mild (<35%) in 18 of 37 (48.6%), average (35–75%) in 11 of 37 (29.7%) and severe (75–100%) in 3 of 37 (8.1%) (Tables 4, 5 and 6).

Reduction of spinal cord diameter was measured in all study patients. Seven of 37 dogs (18.9%) had no reduction of spinal cord diameter in follow-up MR images, 20 of 37 (54.1%) had a mild (0.75–0.95), 9 of 37 (24.35%) had a moderate (0.35–0.75) and 2.7% (1/37) had a severe (0–0.35) spinal cord diameter reduction (Tables 4, 5 and 6).

**Fig 3:**
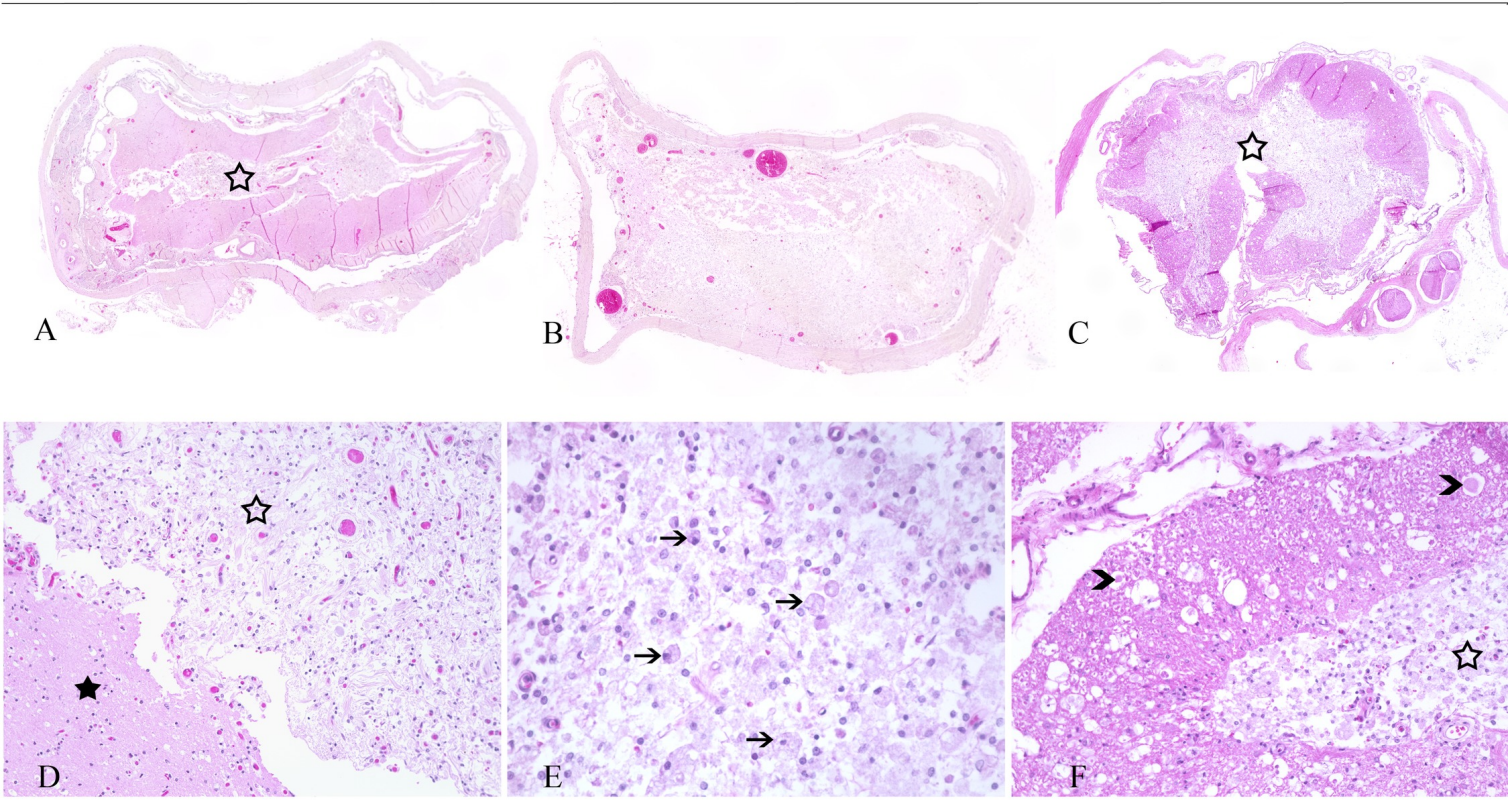
Overview of haematoxylin and eosin-stained sections of the spinal cord epicentre of the 3 dogs with severe, chronic spinal cord injury. Patient ID 7: severe myelomalacia, especially prominent in the grey matter (open asterisk) (A). Patient ID 11: severe pan-myelomalacia with complete loss of organotypic architecture and distinction between grey and white matter (B). Patient ID 16: severe myelomalacia with accentuation in the grey matter (asterisk) (C). Patient ID 7: myelomalacia with gliosis in the grey matter (open asterisk), while the white matter (asterisk) is less prominently affected (D). Patient ID 16: grey matter with severe gliosis and infiltration of phagocytic gitter cells (arrows) (E). Patient ID 16: severe malacia with gitter cell infiltration within the grey matter (open asterisk) (F). The white matter exhibits multiple dilated myelin sheaths and several axonal spheroids (arrowheads).

Three study patients (patient IDs 7, 11 and 16) died spontaneously or were euthanised and sent to the Department of Pathology for post-mortem examination. Comparable findings were observed at the spinal cord epicentre of all 3 dogs during pathological examination (Fig 3). Gross findings included adhesion of the spinal cord meninges with the bony spinal canal wall and degenerated intervertebral disk material, respectively (patient ID 7 and 16) (Table 4). Severe extensive (patient ID 11) and mild focal (patient ID 16) spinal cord atrophy with hourglass-like appearance was noted at the spinal cord epicentre and extending caudally. Histological examination of the spinal cord epicentres of all 3 cases revealed myelomalacia with a predominant manifestation within the spinal cord grey matter in patients 7 and 16, and affecting the complete neuroparenchyma in patient 11 (Fig 3) (Table 7). There was marked reactive infiltration of large phagocytic, so-called gitter cells as well as a proliferation of partially spindeloid glial cells, interpreted as gliosis. The white matter of patients number 7 and 16 exhibited varying degrees of multifocal myelin sheath dilatation, occasionally with axonal drop-out, infiltration of myelinophages and multifocal swelling of hypereosinophilic axons termed spheroids. Additional findings included osseous metaplasia of the dura mater, termed pachymeningitis ossificans, in 1 dog (patient ID 7), while dural calcification was noted in another case (patient ID 11) (Table 4). Overall, the histopathologic findings of intramedullary lesions in the epicentre highly mirrored the observations in the MRI of the patients (Figs 2 and 3).

**Table 7:** Histopathological findings in the 3 patients (patient ID 7, 11 and 16).

Patient ID	Gross findings, spinal cord	Histopathological findings, spinal cord epicentre	Additional findings, spinal cord
**7**	Herniated intervertebral disk material with adhesion to the dura mater at the epicentre, discoloration of spinal cord neuroparenchyma with washy demarcation between grey and white matter.	Severe grey-matter accentuated myelomalacia; moderate multifocal infiltration of phagocytic gitter cells and gliosis; moderate multifocal myelin sheath dilatation and occasional myelinophages in all surrounding white matter fascicles.	Mild multifocal osseous metaplasia of dura mater, so called pachymeningitis ossificans.
**11**	Severe extensive atrophy with hourglass-shape of spinal cord.	Severe pan-myelomalacia with complete loss of organotypic structure of the neuroparenchyma and diffuse gliosis.	Mild multifocal calcification of dura mater.
**16**	Mild focal atrophy with hourglass-shape of spinal cord; adhesion between spinal dura mater and bony vertebral canal.	Severe grey-matter accentuated myelomalacia; moderate multifocal infiltration of phagocytic gitter cells and gliosis; severe multifocal myelin sheath dilatation, multiple myelinophages, and axonal spheroids in all surrounding white matter fascicles.	None.

Length of intramedullary lesions and intramedullary cavitations were compared with severity of initial SCI, defined as neurological grade immediately (≤48 hours) after SCI, and hyperintense intramedullary signal in T2WI in initial MRI examinations using Student's t-test. The number of decompressive surgeries and outcomes were also compared with the length of intramedullary lesions/cavitations using Student’s t-test.

**Fig 4:**
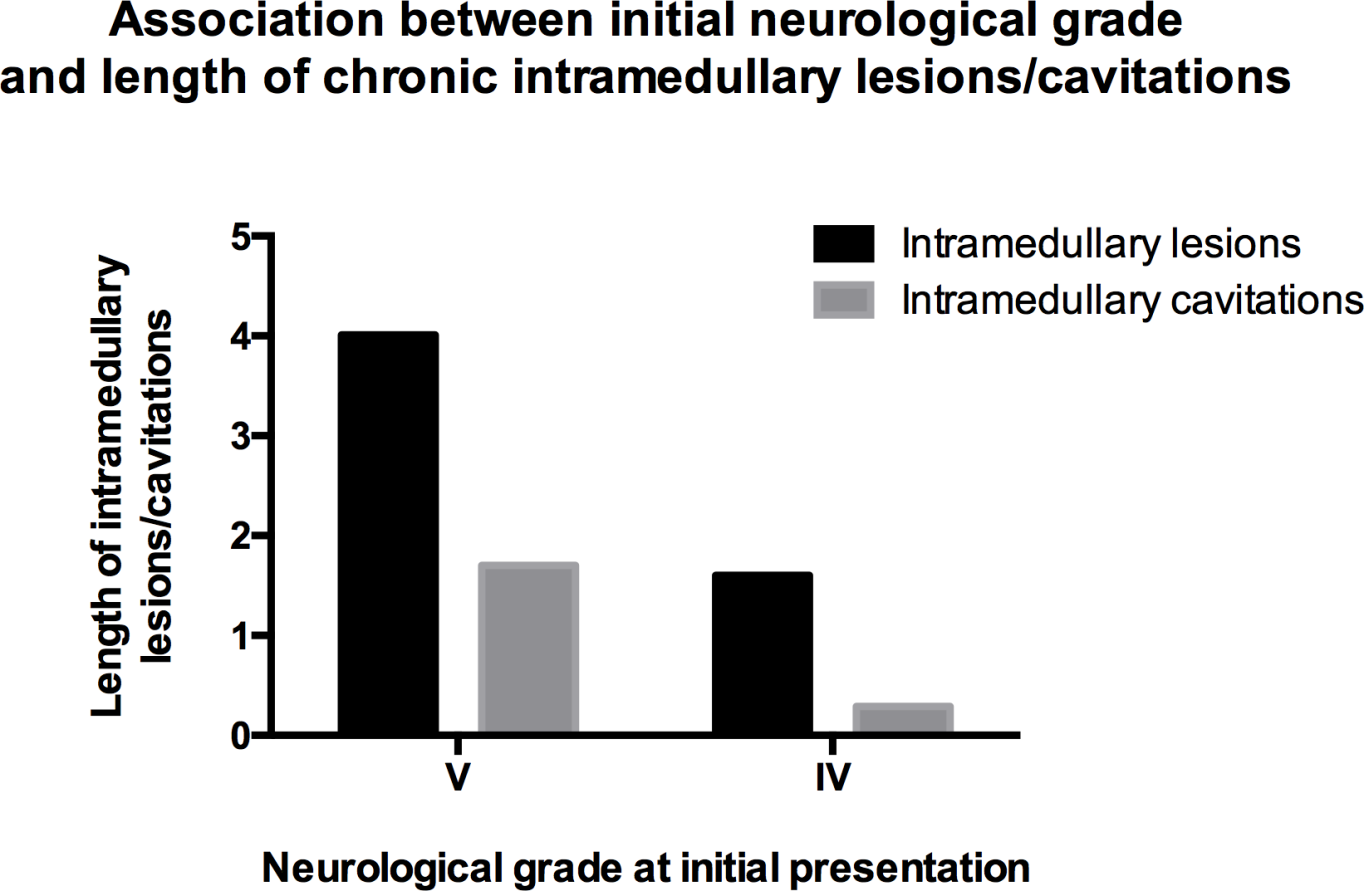
Association between length of chronic intramedullary lesions/cavitations and neurological grade following SCI (≤48 hours). Statistically significant difference between grade IV and V in regard to extension of intramedullary lesions (P = 0.002) and intramedullary cavitations (P = 0.008).

The length of intramedullary lesions and cavitations was associated with the initial neurological status of the study patients. Dogs that presented with grade V were significantly more likely to develop longer intramedullary lesions and cavitations compared to dogs that initially presented with neurological grade IV (P = 0.002 and P = 0.008, respectively) (Table 4, Fig 4).

No association was found between the hyperintense intramedullary signal in T2WI in the initial MRI examination and the length of chronic intramedullary lesions and intramedullary cavitations (P = 0.207/P = 0.149).

Dogs that had 2 decompressive surgeries did not develop longer intramedullary lesions/cavitations compared to dogs that had 1 decompressive surgery (P = 0.383/P = 0.363).

**Fig 5:**
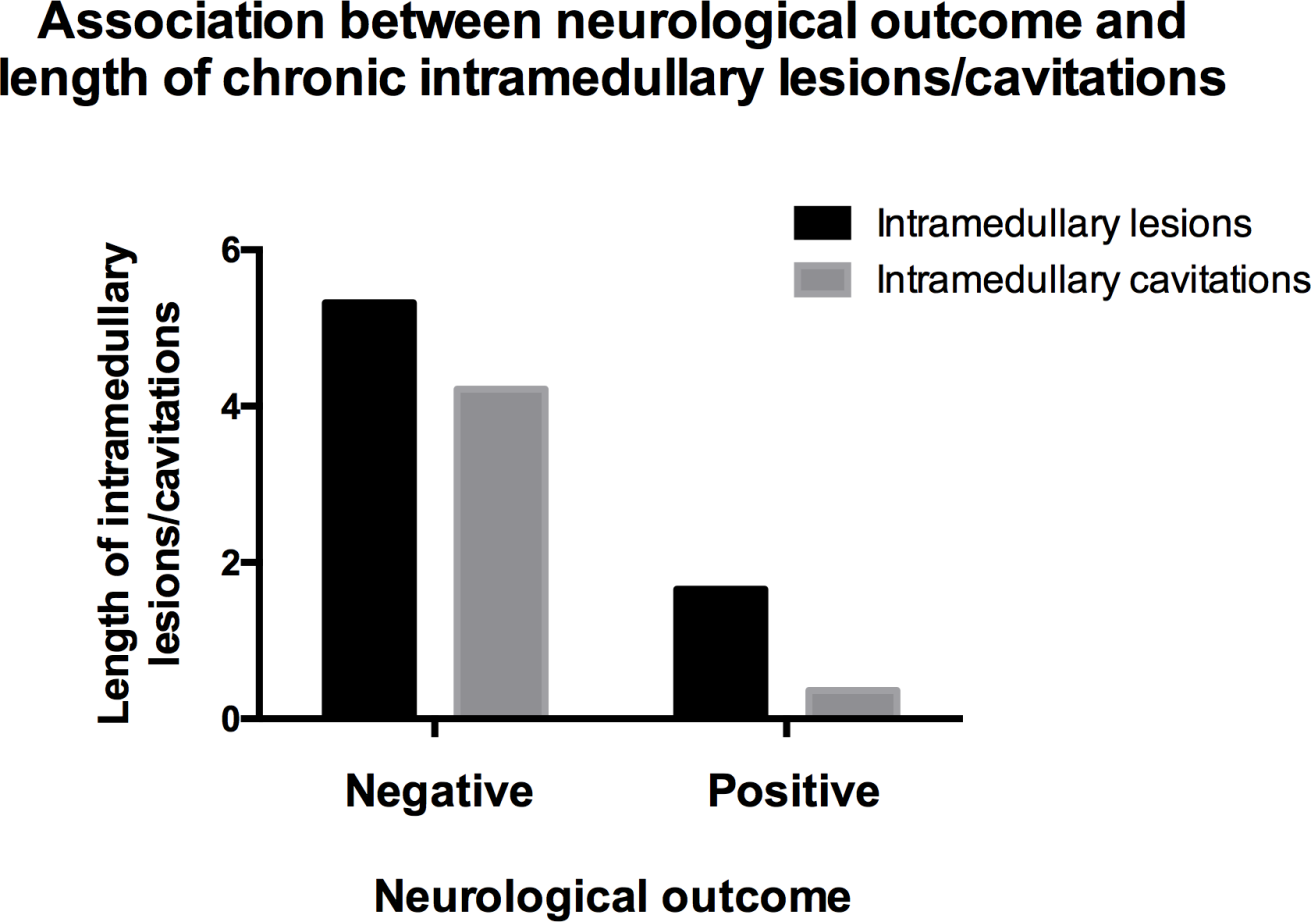
Association between length of chronic intramedullary lesions/cavitations and neurological outcome (P<0.0001). Dogs with negative neurological outcomes did not regain ambulation within 7 months after presentation in the hospital. Dogs with positive neurological outcomes regained ambulation within 7 months after presentation in the hospital.

The outcome was significantly associated with length of intramedullary lesions and cavitations (P<0.0001). A positive outcome was highly associated with shorter intramedullary lesions and cavitations and *vice versa* (Fig 5). Dogs with positive outcome had a median length of intramedullary lesions and cavitations of 1.66 and 0.36 in comparison with L2 vertebra length, respectively (Tables 4, 5 and 6). Dogs with negative outcome had median values of length of intramedullary lesions and cavitations of 5.32 and 4.22, respectively.

Two variable tables were created to test the correlation between the outcome and lesion restriction to the SCI epicentre. Positive outcomes were negatively correlated with intramedullary lesions and intramedullary cavitations, extending beyond the lesion epicentre (weak negative correlation, simple kappa = -0.317/-0.375).

Eighteen dogs with positive outcomes developed intramedullary lesions (Tables 4 and 6). Myelopathies were detected in all available MRI scans 3 weeks following SCI or later. For 5 dogs with positive outcomes, more than 1 MRI examination of chronic intramedullary lesions was available (Table 3). Intramedullary lesions and cavitations became more pronounced in the following MRI examinations in 4 and 3 patients, respectively. In 1 patient a regression of intramedullary lesion was observed, but the cavitation expanded. Intramedullary lesions in dogs with negative outcomes were detected in all MRI scans at all available time intervals after SCI (Tables 4 and 5). Sixteen dogs with negative outcomes had more than 1 MRI performed more than 3 weeks after SCI (Table 3). The intramedullary lesions and cavitations expanded with time in 6 and 7 patients, respectively. In 1 case, cavitation was not detected in the first MRI scan in the time period between 3 weeks and 2 months after SCI, but was present in the following MRI scans.

Eighteen of the 37 study patients (48.65%) developed chronic cavitations, extending beyond the SCI epicentre. These cavitations were considered to be post-traumatic syringomyelias. Post-traumatic syringomyelias were detected in 6 of 21 (28.57%) dogs with positive outcomes and 12 of 16 (75%) of dogs with negative outcomes (Tables 4, 5 and 6).

To find an association between clinical data and mCSA of intramedullary lesions and cavitations, a chi-square test was used.

Dogs initially demonstrating neurological grades IV and V were statistically different in regard to mCSA of intramedullary lesions and intramedullary cavitations found in follow-up MRI studies in their spinal cords (P = 0.041/0.005). Dogs with neurological grade V at presentation had more extensive cavitations, observed in the transverse MRI planes, than dogs with grade IV.

Hyperintensity in T2W MRI immediately after SCI was associated with mCSA of intramedullary lesions (P = 0.004). On the other hand, a hyperintense intramedullary signal in T2WI immediately after SCI was not associated with mCSA of intramedullary cavitations (P = 0.095).

Maximal CSA of intramedullary lesions and cavitations was not associated with number of decompressive surgeries performed (P = 0.323/0.140).

Neurological outcome was significantly associated with mCSA of intramedullary lesions and intramedullary cavitations (P<0.001/P = 0.012). Dogs with positive outcome developed less extensive intramedullary lesions/cavitations compared to dogs with negative outcome.

A chi-squared test was applied to evaluate the association between degree of spinal cord diameter reduction and outcome. Reduction of spinal cord diameter was not associated with outcome (P = 0.078).

## Discussion

In the current study, the MRI features of chronic intramedullary lesions following SCI in dogs were described, and the associations between imaging and clinical data were identified. Thirty-four of the 37 current study patients (91.9%) developed chronic intramedullary lesions, and 32 of 37 (86.5%) developed intramedullary cavitations. In human patients, the prevalence of post-traumatic intramedullary lesions reaches more than 80%, and cavitations (including post-traumatic syringomyelias) reach up to 51% [14, 22, 23]. The prevalence of post-traumatic intramedullary lesions/cavitations is lower in human SCI studies compared to our study. However, in human studies, initial neurological grade after SCI varied from mild to severe. In the current study, all dogs had severe neurological deficits ranging from non-ambulatory paresis to paraplegia, representing severe initial SCI.

In the current study, dogs that presented at the hospital with neurological grade V were more likely to develop more extensive (length and lesion mCSA) myelopathies and cavitations than dogs that presented with grade IV (Fig 4). This supports our hypothesis that more severe initial spinal cord trauma leads to extensive chronic intramedullary changes and cavitations. In human medicine, the presence of severe chronic spinal cord changes, including myelomalacia, spinal cord atrophy, cystic cavities and syringomyelia is also directly associated with severe initial SCI [15, 16]. The risk of development of post-traumatic syringomyelia in complete (absent sensory or motor function) initial SCI is higher than with milder neurological presentation [27, 31–33]. Other authors could not confirm this association [22, 34]. However, in the latter studies, the extent of lesions was not considered (i.e., smaller cavitations restricted to the SCI epicentre were classified as syringomyelias) [22, 34].

All dogs in the current study with hyperintensities in initial MRIs (n = 18) developed intramedullary lesions in follow-up examinations, and 16 of them developed intramedullary cavitations. On the other hand, from 8 dogs without hyperintense signal in initial T2W MR images, 4 developed intramedullary lesions, while the other 4 dogs did not. Although the association between the length of intramedullary lesions/cavitations in sagittal MR images and preoperative hyperintensity in T2WI was not significant, a significant association was found between mCSA of chronic myelopathies and hyperintensities in the initial T2W MR images. Intramedullary hyperintensity in initial T2W MR images following SCI are thought to be associated with spinal cord oedema, contusion, inflammation, myelomalacia and necrosis in the spinal cord in human and canine patients [35, 36]. Initial MRI is used as one of the predictive factors for the outcome in dogs with SCI following acute IVDH. In the study of Ito et al., only 55% of paraplegic dogs with intramedullary hyperintensity following acute IVDH regained ambulation, whereas all dogs without initial T2W intramedullary hyperintensity regained ambulation [25]. Other studies involving dogs with various initial neurological grades only showed a tendency for dogs with intramedullary hyperintensity to have a poorer outcome [26, 30, 37]. A similar prognostic value of MRI for functional outcomes has been described in human patients after spinal cord trauma [38–40].

In the current study, more extensive chronic intramedullary lesions/cavitations were strongly associated with worse neurological outcomes (Fig 5). It is known in veterinary medicine, that extensive syringomyelia can cause chronic pain, ataxia and mild paresis in dogs, but never plegia *per se* [41–43]. We did not observe the formation of syringomyelia being linked to deterioration of clinical signs in dogs, but we speculate that it contributes to the lack of clinical improvement. Studies in human medicine have shown that the presence and extent of spinal cord cavitations/syringomyelia are associated with more severe clinical signs in follow-up examinations and with deterioration of clinical signs [14, 16, 35]. In veterinary medicine, there are limited data comparing outcome and MRI findings of chronic intramedullary changes following SCI. Nevertheless, the search for new treatment strategies in chronic paraplegic dogs is ongoing [18, 19, 44]. In transplantation studies, intramedullary hyperintensities and cavitations in T2W MR images in chronic paraplegic canine patients were detected a few months to several years following SCI [18, 19]. In a study performed by Forterre et al., dogs with positive outcome after IVDH surgery did not have any intramedullary changes in follow-up MRI examinations 12 weeks following acute SCI [24]. However, the initial neurological grade/T2W hyperintensity in the initial MRI examinations of these dogs are unknown, which means that the initial SCI could have been milder than in our study patients. In the current study, the outcome was considered to be positive when dogs regained ambulation, which leads to a certain limitation because rehabilitation time and final degree of paraparesis or ataxia were not considered. A weak correlation was found between location of intramedullary myelopathies/cavitations and neurological outcome of the patients. Lesions restricted to the SCI epicentre were associated with positive outcome, which is in agreement with earlier observations in humans [14, 35].

No association was found between the number of decompressive surgeries and the extent of intramedullary lesions and cavitations in sagittal and transverse MR images. The influence of spinal cord manipulation and spinal canal stenosis on development of different intramedullary changes is still in debate in human medicine [14, 27, 34]. It is strongly believed that scar formation in the dura mater and subarachnoid space may be the key mechanism in the formation of post-traumatic syringomyelia [45–48]. It should be taken into consideration that human SCI is frequently associated with malalignment of the vertebral canal requiring other surgery techniques than the hemilaminectomy normally used according to the standard protocol in naturally occurring IVDH in dogs [28]. The current study is in line with other veterinary field studies, showing a benefit of multiple surgeries for functional outcome if they provide proper spinal cord decompression [24, 49]. One of the limitations of the study is the surgeon’s experience which was not considered. Surgeons’ experience was shown to be associated with neurological outcomes [50].

Thirty of 37 (81.1%) of our study patients had reduced diameters of their spinal cords, restricted to the SCI epicentre. Nevertheless, we could not find a significant association between the degree of diameter reduction of spinal cord and outcome of our patients. A small amount of white matter in the subpial region of the spinal cord in dogs may be enough to preserve motor function [7]. In human patients, spinal cord atrophy in MR images is defined as decreased anteroposterior diameter of the spinal cord, measuring 5 to 6 mm or less [16]. In canine patients, the exact extent has not been clearly defined, most probably because of spinal cord diameter size variation in different breeds. In more than 50% of human patients suffering from chronic paraplegia after spinal cord trauma, spinal cord atrophy is detected and restricted to the injury site [51]. These chronic changes in the spinal cord are not associated with formation of syringomyelia, but they are associated with a worse neurological outcome [16, 52].

One of the limitations of our study was the imprecision of manual intramedullary lesion measurement in the MR images [37].

To the authors’ knowledge, MRI features of chronic intramedullary changes have not been well described in veterinary medicine up to this point. Chronic intramedullary fluid-filled cavitations of our study dogs display the same imaging pattern as syringomyelia described in humans and dogs [13, 14, 16, 20]. Non-cavitational intramedullary changes detected in our patients‘ MR images resemble malacic changes as described in human patients and MRI features of ascending—descending myelomalacia in dogs [12–14, 16, 36, 53]. However, chronic myelopathies following IVDH detected in our patients did not progress rapidly and were found primarily in the grey matter of the spinal cord. In 10 of our 21 patients (47.61%) that underwent more than 1 MRI examination at least 3 weeks following SCI, the intramedullary lesions did not increase with time. Unfortunately, we could not detect any clinical factors that contributed to the expansion of intramedullary lesions.

Histopathological examination of 3 of the study patients revealed severe myelomalacia with a predominant manifestation within the grey matter and variable extent into the white matter of the spinal cord. The degenerative changes were associated with a reactive response of phagocytic cells clearing necrotic debris. Moreover, there was reactive proliferation of partly spindeloid glial cells, most probably indicating reparative attempts in terms of glial scar tissue formation. Two of the dogs additionally displayed a reduced and hourglass-shaped diameter of their spinal cord at the lesion epicentre, thus indicating atrophic changes of the neuroparenchyma (Table 4) (Fig 3). Overall, the histopathological findings highly corresponded to the MRI data (Figs 2 and 3).

Spinal cord cavitations are thought to be sequelae to myelomalacia/necrosis in the spinal cord in both humans and dogs [12, 45]. Initial spinal cord trauma may cause bleeding, contusion or oedema, subsequently leading to local necrosis of the parenchyma. Necrotic tissue is liquefied and replaced with a cavity, which is filled with fluid [12, 45]. In the human literature, it is speculated that cavities without continuous enlargement do not cause neurological problems, but cavities with progression in size are associated with neurological deterioration [14]. Several mechanisms have been suggested to explain the expansion of the cavity, but the intramedullary pulse pressure theory is the most widely accepted. According to this theory, increased pulse pressure in the spinal cord in comparison to the subarachnoid space leads to expansion of syringomyelia [45, 54]. In humans, syringomyelia with a tendency to grow is lined with dense tissue and surrounded by reactive gliosis [55]. In contrast, intramedullary fluid-filled cavitations, that are not associated with clinical signs, mostly have thin astrogliotic walls [55]. In 3 dogs in the current study, the spinal cords of which were investigated histologically, the linings surrounding the malacic/necrotic areas were considered to be rather thin and should represent the cavity without pressure. We speculate that this feature could have varied in different study patients.

## Conclusions

In conclusion, we have confirmed that chronic intramedullary lesions/cavitations mirror the severity of initial trauma to the spinal cord and correspond to the neurological state in dogs. These findings are in line with human SCI investigations [14, 16, 27, 31–33]. The number of decompressive surgeries in dogs is not associated with the extent of intramedullary lesions. This is partly in line with human SCI studies [34]. However, chronic reduction of spinal cord diameter was not associated with a worse outcome in the current study as it has been described in humans [16, 52].

The current study provides evidence that the canine patient with naturally occurring IVDH is a useful model for human SCI investigations.

## Supporting information

S1 Table Clinical and diagnostic imaging information of the study patients. **Number**–patient number in veterinary hospital database.
**Patient ID**–number, corresponding to patient ID number in the manuscript.
**Acute cases (+/-)**—+ patients presented at the hospital in acute phase of SCI (<3 weeks following SCI);—patients presented at the hospital in chronic phase of SCI (>3 weeks).
**Surgery at referring veterinarian (+/-)**—+performed;—not performed.
**MRI time points**– 0 = before decompressive surgery; 1 = 1–3 weeks after decompression; 2 = 3 weeks-2 months; 3 = 2–5 months, 4 = 5–8 months, 5 = 8–11 months, 6 = 11–14 months, 7 = 14–17 months, 8 = 17–20, 9 = >20 months after adequate decompression.
**SCA >3 weeks following decompression**—0 = no atrophy; mild atrophy 1 = 0,75–0,95; average atrophy 2 = 0,35–0,75; severe atrophy 3 = 0–0,35 spinal cord diameter in SCI epicentre in comparison to normal spinal cord diameter.
**Maximal CSA of intramedullary lesion**—0 = no lesion, 1 = 1–35%, 2 = 35–75%, 3 = 75–100% of the spinal cord area.
**T1W MRI examination**—+ available,—not available.
**FLAIR MRI examination**—+ available,—not available.
**Fluid-filled cavitations/syringomyelia—**+ present;—not present.
**Maximal CSA of intramedullary lesion**– 0 = no lesion, 1 = 1–35%, 2 = 35–75%, 3 = 75–100% of spinal cord area.
**Intramedullary hyperintensity in initial T2W MR images (+/-)**—+ present;—not present; n.a. = not available.
(XLS)
